# Single-incision Laparoscopic Appendectomy for acute Appendicitis using a 10-mm Laparoscope and the Glove Port Technique

**DOI:** 10.12669/pjms.332.10022

**Published:** 2017

**Authors:** Takaaki Tsushimi, Hirohito Mori, Manabu Sudo, Yoshihide Minami, Koichi Ueki, Makoto Tamai

**Affiliations:** 1 Dr. Takaaki Tsushimi, Ehime Rosai Hospital, Department of Surgery, Japan. Minamikomatsubara-cho 13-27, Niihama, Ehime, Japan, Saiseikai Shimonoseki General Hospital, Department of Surgery. Shimonoseki, Yamaguchi, Japan; 2 Dr. Hirohito Mori, Ehime Rosai Hospital, Department of Surgery, Japan. Minamikomatsubara-cho 13-27, Niihama, Ehime, Japan; 3 Dr. Manabu Sudo, Saiseikai Shimonoseki General Hospital, Department of Surgery. Shimonoseki, Yamaguchi, Japan; 4 Dr. Yoshihide Minami, Saiseikai Shimonoseki General Hospital, Department of Surgery. Shimonoseki, Yamaguchi, Japan; 5 Dr. Koichi Ueki, Saiseikai Shimonoseki General Hospital, Department of Surgery. Shimonoseki, Yamaguchi, Japan; 6 Dr. Makoto Tamai, Saiseikai Shimonoseki General Hospital, Department of Surgery. Shimonoseki, Yamaguchi, Japan

**Keywords:** SILA (Single Incision Laparoscopic Appendectomy), SILS (Single Incision Laparoscopic Surgery), Appendectomy, 10-mm laparoscope, Glove port technique

## Abstract

**Objective:**

To evaluate the single incision laparoscopic appendectomy (SILA) using existing instruments, the 10-mm laparoscope, and glove port technique.

**Methods:**

SILA was performed on 16 patients (8 male cases, 8 female cases) between June 2012 and September 2015. A 20-mm incision was made in the umbilicus and a wound retractor was placed. A 10-mm trocar for the laparoscope and two 5-mm trocars were fixed to the three fingers of the latex gloves and it was attached to the wound retractor. Another thin forceps were inserted from right low abdomen.

**Results:**

Average age of patients was 32.6 ± 17.7 years. Preoperative average white blood cell was 13,325 ± 4,584 /mm^3^, and average CRP was 1.81 ± 3.70 mg/dL. Preoperative body temperature was 36.8 ± 0.5°C. The mean appendix size was 9.6 ± 2.3 mm and none of the patients had an abscess on preoperative CT. The CT also revealed a fecal pellet in 5/16 (31%) of patients. Mean operation time was 66.4 ± 25.4 minutes, and minimal intraoperative bleeding was observed in all patients. Average hospital stay was 5.3 ± 1.9 days and none of the patients had complications.

**Conclusion:**

SILA using the 10-mm laparoscope and glove port technique may be a safe and feasible operation for mild to moderate appendicitis.

## INTRODUCTION

First reported by Semm in 1983^1^, laparoscopic appendectomy is a popular treatment modality for acute appendicitis because of the ease of appendix identification, less scarring, and better postoperative pain relief compared to open surgery.^2^,^3^ After Pelosi et al. first performed the single-incision laparoscopic appendectomy (SILA)^4^, it has been used increasingly because of excellent cosmetic results compared with the multi-port laparoscopic appendectomy. Since acute appendicitis is prevalent in the younger generation^5^, good post-operative cosmetic results are very important for this age group. However, SILA is technically more difficult than multi-port laparoscopic appendectomy, it may not be used in cases of appendicitis complicated by abscess or perforation. Furthermore, a specific port or 5-mm laparoscope is already commonly used for single incision laparoscopic surgery (SILS). Several tens of thousands of dollars will be necessary in order to introduce a new laparoscope system.

In this study, we aimed to confirm the feasibility of performing SILA using existing instruments, a 10 mm laparoscope system, and the glove port technique.

## METHODS

### Patients

A retrospective chart review was carried out on patients who underwent SILA for acute appendicitis from June 2012 to August 2015 at the Ehime Rosai Hospital in Niihama, Japan and Saiseikai Shimonoseki General Hospital in Shimonoseki, Japan. The study comprised 16 (8 male and 8 female) patients with mild to moderate symptoms and catarrhal or phlegmonous appendicitis. Patients who underwent multi-port laparoscopic appendectomy or open abdominal appendectomy for necrotic or perforated appendicitis were excluded from the study. Patient characteristics (age, sex, body mass index), as well as preoperative- (body temperature, white blood cell counts, CRP, size of appendix, presence or absence of a fecal pellet), surgical- (operation time, appendix removal method, presence or absence of a drainage tube), and postoperative-related factors (length of stay, complications) were retrospectively analyzed.

### Surgical techniques

**Fig.1:**
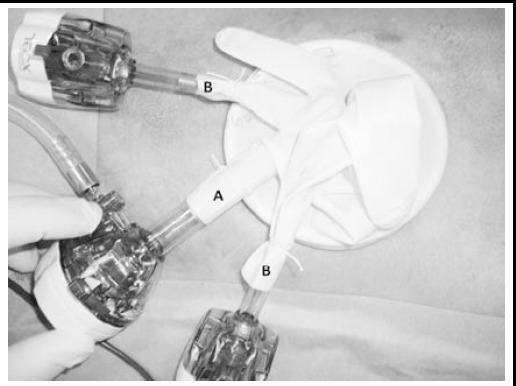
One 10-mm trocar (A) and two 5-mm trocars (B) were fixed to the three fingers of the gloves.

**Fig.2:**
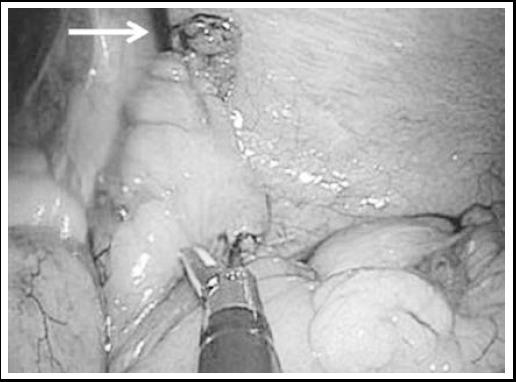
A 2.3-mm thin forceps (white arrow) was used to grasp the appendix and the mesoappendix was cut with ultrasonically activated coagulating shears. One of the two 5-mm trocars was not used in this situation.

**Fig.3:**
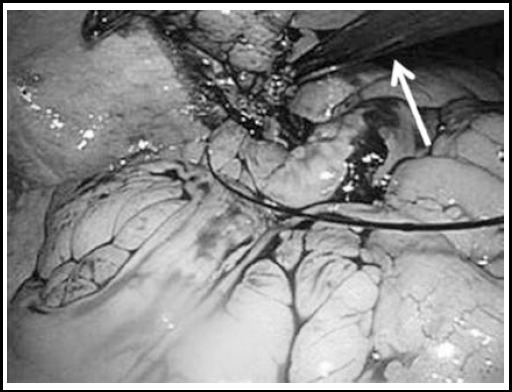
The ENDOLOOP^®^ was guided to the appendicular root by forceps (white arrow) and used for ligation. All trocars (laparoscope, ENDOLOOP^®^, forceps) were used in this situation.

After induction of general anesthesia, a vertical transumbilical skin incision measuring two cm was performed. Upon entering the abdominal cavity, a wound retractor (Alexis^®^ XS) was placed at the umbilical wound. A 10-mm trocar for the laparoscope and two 5-mm trocars were fixed to the three fingers of the size 5.5 latex gloves (Fig.1). The glove with trocars was attached to the wound retractor. The pneumoperitoneum was established by insufflation with carbon dioxide until an abdominal pressure of 10 mmHg was reached. A 10-mm laparoscope was inserted into the abdominal cavity and another 2.3-mm thin forceps were inserted from the right lower abdomen. After visual identification, 5-mm or 2.3-mm forceps were used for grasping the appendix. The mesoappendix was cut with ultrasonically activated coagulating shears (Fig.2). And the appendicular root was laparoscopically double-ligated using an ENDOLOOP^®^(Fig.3). The appendix was either cut using ultrasonically activated coagulating shears and removed, or had its stump inverted with a purse-string suture at the umbilical wound under direct visualization.

## RESULTS

Between June 2012 and September 2015, 8 male and 8 female patients were enrolled in the study. The average age of patients was 32.6 ± 17.7 years and the mean body mass index was 21.1± 4.5 kg/m^2^. The preoperative average white blood cell count was 13325 ± 4584 /mm^3^, and the average CRP was 1.81 ± 3.70 mg/dL. Preoperative body temperature was 36.8 ± 0.5 degrees centigrade. The mean appendix size was 9.6 ± 2.3 mm and none of the patients had an abscess on preoperative CT. The CT also revealed a fecal pellet in 5/16 (31%) patients. The appendicular root was laparoscopically ligated and cut in 9 cases, and was removed and inverted at the umbilical wound under direct visualization in seven cases. Mean operation time was 66.4 ± 25.4 minutes, and there were minimal intraoperative bleeding in all patients. Only one patient (6%) required a drainage tube in the Douglas pouch. Average hospital stay was 5.3 ± 1.9 days. None of the patients developed complications.

## DISCUSSION

Acute appendicitis is one of the most common diseases encountered by surgeons. Although conservative therapy using antibiotics has been widely accepted for acute appendicitis, about 30 % patients eventually received surgical operations within a year.^6^ Thus, appendectomy is still considered the standard treatment for acute appendicitis and has a long history. Since Charles Heber McBurney was the first surgeon who received academic accolades for his expertise in appendicitis,^7^,^8^ his legacy remains in the term “McBurney point,” which refers to the specific point of tenderness or the “McBurney incision,” which is the method used in open appendectomy.

It currently supersedes open appendectomy for acute appendicitis because of obvious benefits, such as relief of postoperative pain, better cosmetic results, and shortened hospital stay.^2^,^3^ Although identification of the appendix may be difficult in open appendectomy due to the small surgical wound in severe appendicitis, or for obese patients, laparoscopic appendectomy facilitates easy identification regardless of physical type or the amount of inflammation. First reported by Pelosi et al., SILA^4^ has been increasing because of excellent cosmetic results compared to multi-port laparoscopic appendectomy.

Acute appendicitis is most common among those between 10-19 years^5^ and cosmetic results are very important in this age group. Nevertheless, SILA generally requires a higher level of technical expertise compared to laparoscopic appendectomy with multi-port because of the scarcity of instruments or the lack of triangulation. Furthermore, SILA is performed using a 5-mm laparoscope or specialized instruments such as the umbilical port.^9^ Additionally, several tens of thousands of dollars are needed in order to introduce a new laparoscope system. Since we cannot afford this additional expense, we thought that we could still perform SILA using our existing laparoscopic system and conventional laparoscopic instruments. Although the glove-port technique takes longer to set up and pneumoperitoneum may suddenly stop when the glove is slipped from the wound retractor, it is very affordable and the technique is applied in various single incision laparoscopic surgeries.^10^ Furthermore, since many commercial SILS ports are made for 5-mm instruments and the glove technique, the 10-mm trocar is easily placed on the finger and is more favorable for SILA using the 10-mm laparoscope. Additionally, since using three instruments (10-mm scope, energy device and laparoscopic forceps) simultaneously from the 20-mm umbilical wound causes significant movement restriction, the appendix was held with 2-mm thin forceps and only the laparoscope and energy device were inserted into the umbilical wound (Fig.2). The umbilical third port was used only if the ENDOLOOP^®^ was guided to the appendicular root or if the intestine was displaced by the forceps (Fig.3).

Many Japanese surgeons invert the appendix stump using the purse-string suture during open appendectomy in order to avoid postoperative residual abscess. Similarly, we also performed this from the umbilical wound and with direct visualization. This technique was utilized among thin patients with an average BMI of 19.8 kg/m^2^. However, all patients who underwent simple ligation without inversion did not present with postoperative residual abscess. We believe that serious postoperative complications in laparoscopic appendectomy did not develop even among those without stump inversions.^11^ Kiudelis et al. did not report any significant differences in intraoperative and postoperative complication rates or hospital stay between patients who underwent laparoscopic appendectomy with ENDOLOOP^®^ simple ligation and stump closure with invaginating suture and those who had open appendectomy, except for the significantly longer operation time in the invaginating suture group.^12^

In conclusion, although we did not include patients with severe appendicitis that was complicated by an abscess or advanced adhesion, SILA using the 10-mm laparoscope and glove port technique may be a safe and feasible operation for mild to moderate appendicitis. We believe that this technique should be aggressively pursued, particularly for younger patients who were not managed in affluent hospital and do not have the capability to buy a new 5-mm laparoscope system.

### Authors’ contributions

***Takaaki Tsushimi and Hirohito Mori*** performed surgical procedure.

***Takaaki Tsushimi*** analyzed and interpreted the patient data and drafted the manuscript.

***Manabu Sudo, Minami, Koichi Ueki and Makoto Tamai*** participated substantially in conception, design, and execution of the study and in the analysis and interpretation data.

All authors read and approved the final manuscript.
